# Socio-cultural beliefs influence feeding practices of mothers and their children in Grand Popo, Benin

**DOI:** 10.1186/s41043-021-00258-7

**Published:** 2021-07-23

**Authors:** Yrence Urielle Amoussou Lokossou, Ayuk Betrand Tambe, Colette Azandjèmè, Xikombiso Mbhenyane

**Affiliations:** 1 Division Human Nutrition, Faculty of Medicine and Health Sciences, Stellenbosch University, PO Box 241, Cape Town, 8000 South Africa; 2 Institut Régional de Santé Publique, Université d’Abomey-Calavi, Cotonou, Benin

**Keywords:** Socio-cultural, Feeding practices, Children and mothers, Benin

## Abstract

**Background:**

Malnutrition is a major public health problem. It contributes to the high death rate among children in developing countries despite the various advocacies of institutions such as WHO and FAO and many other organisations. More research needs to be done in order to contribute to the achievement of the Sustainable Development Goals. The aim of this study was to explore socio-cultural practices and their influence on feeding practices of mothers and their children in Grand Popo, Benin.

**Methods:**

A qualitative research methodology was used with an inductive approach. A pretested discussion guide was used to conduct focus group discussions with participants in their local language. Four focus group discussions were held in 4 villages located in both the rural and the semi-urban areas with each focus group comprising seven to eight participants. The study protocol was approved by the Health Research Ethics Committee of Stellenbosch University. Focus group discussions were recorded, transcribed and translated to English. The data was analysed following the Creswell data analysis steps.

**Results:**

All the children were breastfed, and 56.1% of children under 6 months received breast milk exclusively. Children were introduced to family foods at 5 months with very low consumption of animal protein and fruits. Mothers and children had monotonous diets with high consumption of vegetables and maize-based meals. Food taboos, particularly during pregnancy, were revealed. Those cultural beliefs were still followed by some mothers, and food rich in nutrients were pushed aside.

**Conclusions:**

There is a need for educational interventions to raise awareness of the negative impacts of some socio-cultural practices on the health of the mother and child.

## Background

Malnutrition continues to be a major health burden in developing countries. Globally, it is the most important risk factor for illness and death. Every country is facing a serious public health challenge relating to malnutrition [1]. According to the World Health Organization (WHO) statistics, nearly half of all deaths in children under the age of five are attributable to undernutrition [2]. Between 2000 and 2016, the prevalence of stunting globally declined from 32.7 to 22.9%, and the number of children affected fell from 198.4 million to 154.8 million [3]. Progress is being made to eradicate malnutrition, but authorities remain rather unhurried while undernutrition continues to wreak havoc among children and adults who were overweight, and obesity is increasing. Many studies have been carried out on malnutrition, and despite the various advocacies of institutions such as WHO, Food and Agriculture Organization (FAO) and many other organisations, more work needs to be done to achieve the Sustainable Development Goals (SDGs).

Poor nutrition in the first 1000 days of a child’s life can also lead to stunted growth, which is irreversible and associated with impaired cognitive ability and reduced school and work performance [4]. Rapid growth during pregnancy, breastfeeding, and dietary diversification in the 1- to 3-year period lead to specific nutritional needs during each of these stages [5]. It is crucial to ensure access to optimal nutrition for each mother and child for the first 1000 days of a child’s life. The period of pregnancy and lactation and the first 2 years of life is a special nutritional challenge for those in need of nutrition or with inappropriate feeding practices [6].

Exclusive breastfeeding is defined as the practice of only giving an infant breast milk for the first 6 months of life (no other food or water) and has the single largest potential impact on child mortality of any preventive intervention [7]. Early initiation and exclusive breastfeeding for 6 months provides essential, irreplaceable nutrition for a child’s growth and development and therefore prevent stunting [8]. It serves as a child’s immunisation, providing protection from respiratory infections [9], diarrhoeal diseases and other potentially life-threatening ailments [10, 11]. Exclusive breastfeeding has a protective effect against obesity and certain non-communicable diseases later in life [9]. Globally, 40% of infants between 0 and 6 months old are exclusively breastfed [2]. Over 800,000 children’s lives could be saved every year among children under 5 years if all children 0 to 23 months were optimally breastfed. Breastfeeding improves intelligence quotient (IQ) and school attendance and is associated with higher income in adult life [9]. According to the Demographic and Health Survey (DHS) in Benin, 94% of children under 2 years of age were breastfed, but only 33% were breastfed exclusively until 6 months [12]. On 12 September 2011, the Republic of Benin joined the SUN Movement. At the time, Benin had established a multi-sectoral, multi-stakeholder platform, the National Council of Food and Nutrition under the office of the President. Benin also had a policy document, the Strategic Plan for Food and Nutrition Development which laid out both nutrition-specific and nutrition-sensitive approaches to improving nutrition [6, 12].

Young children need adequate dietary intake (through exclusive breastfeeding followed by quality complementary feeding) to support the rapid rate of growth that occurs in the first 2 years of life. Inadequate feeding and care practices often lead to a rapid decline in nutritional status after birth, and more prominently after 3 to 4 months of age (when other foods beyond just breast milk are typically introduced).

Different forms of taboos and cultural beliefs about food exist. They vary from one society to another and range from food for the adult and food fed to children. For example, snails and cane rat meat are taboo among pregnant women and eggs among children in Southeastern Nigeria [12]*.* Similarly, in rural Ethiopia, pregnant women avoid eating green leafy vegetables, yoghurt, cheese, sugarcane and green pepper as habitual in fear of obstetric complications associated with the delivery of a bigger infant [13]. A study conducted in the Gambia reported that taboos, customs and beliefs contribute to malnutrition among the Fula in different ways [14]. Another study in Kenya also observed that food taboos are delaying the progress in fighting undernutrition because of cultural beliefs [15]. These beliefs are thought to limit the intake of essential nutrients. Furthermore, a study conducted in Papua New Guinea has shown that many foods that are rich sources of protein have been enlisted as taboos for pregnant women. It is believed that because protein helps the body to grow, if a woman consumes a lot of protein in her pregnancy, then the baby will grow too big leading to complications during labour [16]. It is now believed that some of the food taboos on restrictions on what women could eat are rooted in the patriarchal philosophy of the past. It was hypothesised that there is a positive relationship between socio-demographic, nutritional status of children and mothers, dietary patterns and socio-cultural practices. Thus, the aim of this study was to explore socio-cultural practices and their influence on feeding practices of children and their mothers in Grand Popo, Benin. The nutritional status of the children and their mothers has been reported [17].

## Methods

### Methodological design

The study design was exploratory, descriptive and qualitative with an inductive approach [18]. This methodology eased a better understanding of the dietary socio-cultural practices among mothers. The focus group technique was used to collect data.

### Study setting and population

Benin is a French-speaking West African nation with an estimated population of 11.8 million inhabitants. The population of the community of Grand Popo is estimated at 57,636 inhabitants, 28,237 men and 29, 399 women, according to the national demography in 2013 [19]. The community of Grand Popo is in the southwest of the Mono Department. The Mono Division is one of the 12 divisions located in the southwest of Benin. It is bounded to the north by the communities of Athiemé, Comé and Houéyogbé; to the south by the Atlantic Ocean; to the south-west by the communes of Ouidah and Kpomassè; and to the west by the Republic of Togo.

The sample for this study was purposively recruited from participants of the related study from the same community which had 408 mother-child pairs from 24 villages. The children were under 5 years, and if the mother had more than one child, the youngest was preferred for the study. The inclusion criterion was parents living in the area for at least 6 months with children between 0 and 60 months, while the exclusion criteria were households not residing in the areas or those with no children in the 0 to 60 months age range.

Focus groups were held in 4 villages selected from the 24, with eight to ten participants. A common guideline for focus group research is to conduct at least two focus groups for each demographic stratum in the study [20, 21]. For this research, four groups were deemed sufficient to be able to reach data saturation [22].

### Data collection procedure

The four focus group discussions (FGD) were held between February and April 2019. The discussion groups comprised seven to eight participants gathered in the public square or under the tree. Focus groups were conducted to establish the socio-cultural practices that influence children’s diets. The researcher welcomed the participants, asked each of them to introduce themselves and summarised the purpose of the discussion and informed the members that participation was voluntary. They were informed that the discussions were recorded, and confidentiality was assured to them. The researcher facilitated the focus group using the discussion guide which elaborated on specific topics identified to help manage the discussion. Themes were about healthy diet, breastfeeding, practice of exclusive breastfeeding, food usually given to a child in a day, foods necessary for child growth, first year feeding practices, care practices and feeding during illness, food distribution in the household (who is served first), foods taboos, restrictions during pregnancy and breastfeeding (women), foods eaten especially during pregnancy and lactation for mother and child good health, foods young children cannot eat and foods not eaten during illness. Two research assistants, who were students from the Department of Nutrition of the University of Abomey-Calavi, Benin, were selected to help the researcher with information recording. Assistants were observers during the discussions, who recorded and took notes. The researcher facilitated the sessions and allowed participants to express themselves freely.

### Trustworthiness and quality control

Quality control of the focus group guide was assured by choosing guidelines for the discussion from instruments that have been validated in preceding studies [23, 24]. In addition, the guidelines for the FGD were tested in a pilot study in 2017 to assess if the questions were appropriate to be used in the study setting. The discussion guide consisted of six main themes of questions with probing questions that were used when needed. If a new probing question was added during a discussion and provided additional insights, it was then used during the next discussion. The discussion was conducted in the local language (*Mina*) using the French guideline, since most mothers did not understand French nor English. Two research assistants assisted with the recording of information and the capturing of the socio-demographic characteristics of the mothers. The recording was in the local language while the notes were done in French and English.

### Ethical considerations

Ethical clearance was obtained from the Health Research Ethics Committee of Stellenbosch University (Reference no: S16/10/211). The study proposal was also submitted, and administrative authorisation obtained from the Departmental of Health of Mono-Couffo (Benin) to conduct the survey in the region. The recruitment of participants started with the researcher and assistants’ introduction to the chief of the village, where they explained the purpose of the study and sought permission. The chief helped the team to contact and recruit households. Prior to the interview, the mothers were informed of the goal of the study in the local *Mina* language before they were invited to participate in the study and informed consent obtained. The mothers were advised that their participation was voluntary and that they could withdraw at any time point without any explanation. Confidentiality was assured to them. The mothers of the children who agreed to participate in the study were presented with the consent form for signing.

### Data analysis

The focus group data interviews recorded were transcribed and translated from *Mina* and French to English. The data collected was classified and organised into themes before generalisations were made. The analysis was done according to Creswell’s [25] five steps of qualitative data analysis. The data was first grouped into 13 discussion topics (used as codes and focus groupings). After responses were analysed and synthesised in terms of nutritional content and similarities, they were categorised according to the following themes relevant for feeding of a young child while keeping the focus groups: (i) healthy diet, (ii) exclusive breastfeeding, (iii) first year diet and (iv) growth foods.

## Results

### Socio-demographic characteristics

Four focus group discussions were carried out with an average of 8 mothers per focus group (see Table 1 with focus group information).

**Table 1:** Focus group characteristics

Groups	Number	Age range
One	8	20 to 60
Two	7	19 to 40
Three	8	20 to 40
Four	8	22 to 40
Total	31	

A total of 31 mothers between 19 and 60 years selected in both rural and semi-urban areas in Benin participated in the study. These 31 mothers were part of the sample of 408 mothers whose demographic data is reported elsewhere [17]. The majority (93.1%) of the mothers were married or living with the partner and living in a monogamous household. Most of the mothers had no formal education (40.2%) or just the primary level of education (36%). A greater proportion of the households used paraffin oil for lighting and firewood for cooking meals. They got drinking water from manual pumps (39.7%) or wells (44.4%). Few households still used surface water (rivers, ponds or lakes; 5.1%). Regarding sanitation, most (77%) do not have access to toilet facilities.

Few mothers were head of household, and most of them were divorced or widowed. A greater proportion of mothers had income from small shops or depended on the husband. In most households where the participants lived, men decided how to spend money and purchase food, and sometimes, partners decided together. Regarding the economic characteristics of households, 91.7% of households had a monthly income of US$60 and spent on average US$24 + US$11.2 on food [17].

Various studies have indicated that low household income has a direct impact on the nutritional status of children [26–28]. Choosing to buy foods depends on the father; there is thus a risk of malnutrition since women can supply more nutritious food to the children when they control the variety when purchasing food. Most of the households lived in one room, and most of the time, it is a family house.

### Focus groups discussions

Through the focus groups conducted, mothers’ knowledge about nutrition, care practices, food distribution within the household and food taboos were discussed (see Tables 2, 3, 4 and 5). During focus group discussions, the mothers highlighted food taboos practised in their communities, as well as some perceptions and misconceptions about child feeding. Several reasons were given to explain why they avoided certain foods during pregnancy and refrained from feeding to their children. Their verbatim responses are organised in Tables 2, 3, 4 and 5. Their responses are grouped according to themes: exclusive breastfeeding, healthy diet, first year diet, growth foods, care practices, food distribution and food taboos. The interpretation of the FGDs is summarised below.

**Table 2:** Focus groups discussion concerning a healthy diet, exclusive breastfeeding

	Focus group 1	Focus group 2	Focus group 3	Focus group 4	Interpretation
**Exclusive breastfeeding**	- One mother did it. But it was difficult because of environment. Most of mothers give water or porridge to children and old women don’t allow young to do it. - Nurses taught them about it but they think baby is thirsty and need water even the same day of birth. - To do it you need to be strong in your mind and must have milk in your breast.	- What we eat is found in our milk, so the breastfeeding is good. All we breastfeed at least until 18 months. - I heard that milk contains lot of water for example 10ℓ of milk contains 9ℓ of water and 1ℓ of vitamins so if you give water to the child, he will not have many vitamins - Milk alone is not enough for the child, so I give him water. - When we go to the hospital, we received advice, but we all give water to children. They are thirsty and cry to ask for it. If you are with your grandmother, she will say to give it. - Some of mothers give up at 18 months others 24 months, depend on each mother’s feelings	- Breastfeeding is essential. Milk is for child. - Give up at 18 months or 2 and a half years. - My first child had drunk formula because I had a problem with my breast but the second one drank my milk. - No exclusive breastfeeding because child is thirsty, he needs water. It’s hot it’s not easy for an adult what to say about child who cannot tell you if he is not fine. He cries and we give water. - They said breastfeeding exclusively is good but needs of the child are different. - Give water at 3 months. - For myself I wanted to give her milk alone, but his grandmother gave him. When I refused, she took him herself to give it to him. She did it the same day at her birth. Heat water to sterilise it and give it.	- Breast milk is good for children. It develops strength and intellect. They take up to 2 years. It’s just knowledge. Doctors have taught us to give milk to children without water until 6 months. - Milk make children thirsty so we give water. - Breastfed exclusively up to 6 months after I make him imported porridge flour. But this child refuses porridge he prefers dough, rice. - Not exclusively breastfed my child. Weaning at 9 months with maize roasted flour.	Most participants seem to have breastfed their children for up to 18 months. Most said they gave water to the child before 6 months, thus not truly exclusive breastfeeding. Environment, time and misconceptions, and beliefs are limits for exclusive breastfeeding practice.
**Healthy diet**	- See by the physical health of the individual - Eating spaghetti, maize paste, fresh fish sauce, vegetables, *egoussi* sauce (squash seeds) - Food consumed quality - When you are fine, you eat well - Cook food rich in nutrients	- Maize foods, rice, cowpea, egg, peanut - Maize stiff porridge has lots of vitamins. We eat at least once a day. - Eating food that make us feeling good.	- Eating paste and bitter leaves sauce, palm nut sauce, wild basil sauce, *gari* - What you eat, and you feel good that is eating well - A food that you prepare carefully, eat and that’s give you nutrients and good health. Those nutrients your child receives through the breastmilk is a good diet.	- Eat good food. Cowpea, potato, *moringa* it is good for children’s health	Starch and legumes were cited by most groups as part of a healthy diet. The feeling of eating food you appreciate and that one assumes nutritious form part of a healthy diet.

**Table 3:** Focus group discussion concerning the diet of the child’s first year and growth foods

	Focus group 1	Focus group 2	Focus group 3	Focus group 4	Interpretation
**First year diet**	- Infant formula milk. - First food to give to child after milk should be cereals. - At 3 months, some start giving simple maize porridge or maize roasted porridge. - Others make flour with maize, soybean (roasted), biscuit, small dried fish (*dowevi, tcheke*) and grind it in small portion to see if infant like it. - Use also sorghum and maize, do not like soybean because it is not good for all children. - Can buy the porridge in the supermarkets. - Rice and macaroni.	- We feed child when we eat giving him portion by portion. - We start with maize porridge (flour and water) maize dough and jute sauce, macaroni to give it the taste of family meals and after it will be used. - You can give him egg sometimes or donut when he’s on the back, so he sucks or eats. - It depends on the child and how he accepts eating.	- At 6 or 7 months, I start with porridge. - Eat the usual foods. - No time to give food especially. He will be taken off breast and when I want to eat, I also give him if he could eat. - Maize porridge, paste and jute sauce, rice, macaroni, cowpea no egg frequently it is when we have money, we buy it. - Maize porridge with maize flour and water. - Porridge and cow milk. I no longer give egg because I cannot afford to buy. We give fish and meat if present in the sauce.	- Macaroni, paste and jute sauce, rice, cowpea. - Child can eat cowpea at 10 months. - He couldn’t. He is too young for that. - He could. Just prepare it well and crush. Give meat and fish occasionally. Eat eggs regularly, at least three times a week.	Most mothers cited maize porridge as the first food followed by other foods or what the family eat. Their responses confirm the types of foods identified as regularly consumed.
**Growth foods**	- Meat, fish, cowpea not too much oil, milk, eggs. - If it is only *gari* and fresh fish you will give to your child, he won’t grow. - Can also prepare vegetable sauce (African eggplant sauce) and put moringa in all foods.	- Cowpea, tilapia, avocado, banana, orange, fruits, vegetables - Just dough we give to our children, and they grow up well. - We do not have processed milk for children. We only have our foods here. When one gives small fish, someone thinks that it is poverty.	- Paste, cowpea, macaroni, rice, okra and jute sauce, orange, fruit, banana, pineapple. - Pineapple is not good for kids it makes you lose weight is better for adults.	- Fish, vegetables, paste, jute sauce and palm nut sauce	The participants identified protein sources especially fish, legumes, fruits and vegetables as growth foods. They know that common traditional foods can affect growth in their children.

**Table 4:** Focus groups discussion concerning care practices and food distribution

	Focus group 1	Focus group 2	Focus group 3	Focus group 4	Interpretation
**Care practices**	- No special food. - Porridge most of the time. - Treatment at home until situation become worse. - Use herbal tea. - Tablets.	- If you have money you can go to the hospital otherwise you start treatment at home first with herbal teas or go to the healers. - It is more often treated at home first. - When it takes too long time for healing we go to hospital. - We give the usual food but more often porridge because at this moment child don’t want to eat. We give him food if he doesn’t like we try other things. - If it is the measles, we don’t give peanut. He does not wear clothes.	- Give mostly porridge, and some others like rice, paste if he likes. - We treat at home first, hospital is expensive. Go there when it is very serious and has worsened. - We give tablets we have at home. - We prepare herbal teas. Grandmothers show us leaves and we prepare. We give as drink or wash them with. The fever goes off quickly.	- We treat at home first we prepare herbal tea if after three days he is still sick we go to the hospital. - He eats usual food and what he likes. When they are sick, they don’t take foods too fat. It is not good. - When they cough or have digestive problems, they cannot eat okra.	Most mothers said they treat their children at home using tablets or herbal tea and only go to the hospital when the condition worsens. They also said that they get or listen to the advice from the children’s grandmothers on how to care for their children. They primarily give maize porridge to the child during sickness and when he/she gets better, normal family food is given.
**Food distribution**	- Children received first and then men are served. - As soon as you finish preparing you serve them.	- Children are served first immediately you finish cooking. - Fathers are outside they may have found something to eat outside, and child would be fasting at home? Children are served first. - They are served first but the large portions of meat or fish are reserved to fathers.	- Dad is served first. Dad’s portion is first removed after cooking. - It is children who is served first. They are hungry and need more food than adults. But it’s for dad the best part, the big fish/meat. - Serve my children first after their father and me.	- Children are served first. - Children are served first and after men are served but the good portion of fish or meat is reserved to the father.	Most mothers said children are served first, but the men receive the ‘best’ part of the meals, and in some households, the men are served first, or their portions are removed before serving the children and other members of the household.

**Table 5:** Focus groups discussion concerning food taboos

	Focus group 1	Focus group 2	Focus group 3	Focus group 4	
**Taboos**	- Our community does not eat crabs, catfish that families do not take. There are family totems. - The egg is not good when you are pregnant because you can quickly give birth. - When you breastfeed, you do not eat African eggplant, mango. They give digestive problems.- Don’t give eggs to child frequently. When you have sometimes you can give him if not it is not possible because too expensive. The reason you have poultry is for herd increasing and sell it after chicken.	- Not everyone has taboos. - If I eat something and I do not digest well, I do not eat anymore. I don’t have taboos, do not eat catfish. - Do not eat catfish we do not eat pork, dog, cat, snake meat in my family but I eat as soon as I find. - When we are pregnant, we do not eat cane rat (giant rats). - We do not eat wild meat, so children do not become violent and behave like a wild animal. - We do not eat local eggs because you can have a dead child because they will not be glad that you take his progeny. We can eat imported eggs. Also, kids can break things like hen so it’s best not to eat them. - We drink maize porridge and dough to have lot of milk.	- Do not eat African eggplant sauce that gives digestive trouble and diarrhoea. - No eggs during pregnancy. We can eat but whole, we couldn’t share with someone else. - A pregnant woman should not prepare eggs and give to someone. She must cook for herself and when she removes the shell it should not have any hurt otherwise, she should not eat because she might have birth complications. - Pork is taboo for Muslims and some families. - Dog, cat, catfish, jute sauce with palm oil. There are no explanations, we got it from ancestors. - No taboos only dog and pork meat if not I eat everything. But when pregnant I do not like anything.	- Pork and dog are my family totem. - Sheep. - Varanus, crab, anyone in this community don’t eat catfish. - Pregnant I do not eat fish it gives me nausea just the paste and chilli. - Pregnant I eat everything. It is said that we should not eat eggs to avoid birth complications. - When we are breastfeeding, it is recommended to eat maize dough, African eggplant, Bitter leaf, maize fermented porridge. - For some, they think if you give rice or cowpea to child, he will have digestive problems, but it depends on what you teach to your child. - When breastfeeding, we do not eat banana, mango, papaya to avoid colic in children. - We eat a lot of *moringa*. We were visited by people who taught us that it is good for children.	Catfish, crabs, eggs, pork and African eggplant were cited by many as taboo foods, especially for pregnant women. Some foods which are rich in nutrients are forgotten because of cultural or religious beliefs.

#### Exclusive breastfeeding

Most participants seem to have breastfed their children for up to 18 months. Most said they gave water to the child before 6 months, thus not truly exclusive breastfeeding. Environment, time and misconceptions, and beliefs were limits identified to influence exclusive breastfeeding practice.

#### Healthy diet

Starch and legumes were cited by most groups as part of a healthy diet. The feeling of eating food you appreciate and that one assumes nutritious form part of a healthy diet.

#### First year diet

Dietary practices during the first year revealed that most mothers cited maize porridge as the first food followed by other foods or what the family eat. Their responses confirm the types of foods identified as regularly consumed in other studies.

#### Growth foods

The participants identified protein sources especially fish, legumes, fruits and vegetables as growth foods. They knew that common traditional foods could affect the growth of their children.

#### Care practices

Most mothers said they treat their children at home using tablets or herbal tea and only go to the hospital when the condition worsens. They also said that they get or listen to the advice from the children’s grandmothers on how to care for their children. They primarily give maize porridge to the child during sickness, and when he/she gets better, normal family food is given.

#### Food distribution

Most mothers said children are served first, but the men receive the ‘best’ part of the meals, and in some households, the men are served first, or their portions are removed before serving the children and other members of the household.

#### Food taboos

Catfish, crabs, eggs, pork and African eggplant were cited by many as taboo foods, especially for pregnant women. Some foods which are rich in nutrients are forgotten because of cultural or religious beliefs.

## Discussions

### Mothers’ knowledge and perceptions about nutrition

This section covers responses on the themes of exclusive breastfeeding, first year diet, healthy diet and growth foods. The verbatim responses are summarised in Tables 2 and 3. The results showed that mothers had limited knowledge about nutrition. For the majority who did not go to school, the source of information remains the elders and the cycle is perpetuated. Despite some nutrition education sessions during hospital care visits, mothers do not apply the knowledge learnt and recommendations proposed by the health workers, either because they say they do not have time or because their family environment does not allow for it. For example, in focus group 3, the mother had the will to exclusively breastfeed her child, but the mother-in-law refused and gave water to the child from the first day. She said: ‘For me, I wanted to give him milk only, but his grandmother gave him water. When I refused, she took him herself to give it to him. She did this the same day of his birth’. The belief that breast milk alone is not sufficient in meeting the nutritional needs of infants remained persistent and as soon as the child cried, he received water. One of the mothers said: ‘No exclusive breastfeeding because child is thirsty he needs water. It’s hot it’s not easy for an adult what about a child who cannot tell you if he is not fine. He cries, and we give water’. Another one said: ‘Babies are thirsty and cry to ask for water. If you are with your grandmother she will say give it’. The findings indicate that mothers are sometimes negatively influenced by significant grandmothers and others as to how to practise breastfeeding [29]. Most mothers seem to have breastfed their children for up to 18 months, according to the information provided during the discussions. However, most revealed that they gave water to the child before 6 months, thus not truly exclusive breastfeeding according to the WHO definition [30]. Environment, time and misconceptions, and beliefs are determinants for exclusive breastfeeding practice that were distilled from the conversations (Table 2). Our findings are supported by other studies which showed that the practice of exclusive breastfeeding and early breastfeeding does not seem to be limited by mothers’ knowledge but by socio-cultural representations in certain societies. In Senegal, 42% of children received water before 6 months [31]. In South Africa, Seonandan and Mckerrow [32] showed in their review on infant and young child feeding practices in a hospital and some homes in KwaZulu-Natal Midlands that 76% of infants were ever exclusively breastfed with just 36% being exclusively breastfed beyond 3 months. Many children receive other fluids other than breast milk even before 1 month. One study in Western Cape showed that 90% of the mothers had introduced water, and 83% of them did it before the age of 1 month [33]. There is a void between recommendations and practices, and findings from this study point to socio-cultural determinants.

Social norms have a great impact on the quality of care provided to children by their mothers. There is a saying in West Africa that the whole family is responsible for supporting the child while growing up. The first year of a child’s life is a delicate time that all mothers recognise. They introduce the child to adult food from as early as 3 months, or later, 5 or 6 months. On the topic of first year diet, most mothers cited maize porridge as the first food followed by other foods or what the family eat (Table 3). Their responses confirm the types of foods identified as regularly consumed. These foods included cereal porridge, which is mostly derived from maize. A simple corn meal is given to the child several times a day, in addition to breast milk. This porridge is low in protein and fat and the child would therefore require additional sources of these nutrients. A South African study has recorded several dietary deficits and a rising trend in the consumption of inappropriate nutritionally poor food during the first year [34]. Budree et al. [35] dedicated a high daily consumption of processed meat (56%) and inappropriate foods such as fruit juice (82%), soft drinks (54%) and refined sugary foods (51%) at 1 year of age. In another study done in Benin [36] cereal porridges which were unenriched were recorded as complementary foods given to children over 6 months and low consumption of fruits and eggs. Some affordable porridges for complementary feeding have been formulated to fulfil the nutritional needs of children from 6 months and older [34, 37]. A mother from focus group one stated that: ‘Others make flour with maize, soybean (roasted), biscuit, small dried fish (dowevi, tcheke) and grind it in small portion to see if infant like it’. In focus group 2, one said ‘We start with maize porridge (flour and water), maize dough jute sauce and macaroni to give the child the taste of family meals and after that the child will be used to the taste’. Studies in West Africa have shown that the diet of many households is composed of cereals, roots, tubers and root products, legumes and nuts, eggs, fish, vegetables and oil [37, 38]. Zeba et al. described dietary patterns in Ouagadougou’ adults in Burkina Faso as high in local cereals, legumes and traditional green leafy vegetables [38] while Frank et al. [39] characterised the urban Ghana dietary pattern as high intakes of plantain, green leafy vegetables, beans, egg, fish, maize, palm oil, okra and fruits. Despite these practices for adults, mothers tend to give mostly *Owo* (stiff porridge) accompanied by local vegetable soup to the children who are on the family diet. Their diet is in most cases totally lacking in animal protein and fruit sources. Lack of funds and cultural believes limit the consumption of animal protein. Some mothers in focus group four said ‘Child can eat cowpea (vegetable) and jute sauce at 10 months’ another contradicted by saying ‘He couldn’t. He is too young for that’ and another backed the first statement by saying ‘He could. Just prepare it well and crush’. This is an indication that mothers have different perceptions and knowledge on what and how to prepare foods to give to the infant. On protein foods, one mother said ‘Give meat and fish occasionally. Eat eggs regularly, at least three times a week’. The main source of animal protein in Grand Popo, Benin, is fish. This is understandable considering that it is a fishing community, and fish is available permanently in the households. In low-income countries, meat and milk products are often lacking in the diets of children. Yet, the benefits of their consumption especially meat has been shown that it improved growth indicators, micronutrient status and cognitive performance [40]. The low consumption of meat can be explained by household income, accessibility, religious and cultural beliefs as seen in this study. Meat consumption is just occasional at events or parties in this study.

In addition, the consumption of fruits was limited in the present study, no mother mentions fruit or fruit juice as foods given during the first year. Several national and sub-national surveys have demonstrated the low consumption of fruit and vegetables in West Africa [41, 42]. Income, non-availability, non-affordability and access determine the consumption of fruit and vegetables [43].

Concerning a healthy diet, one of the mothers said that: ‘Healthy diet is seen by the physical health of the individual’. Another one said that: ‘Eating imported/processed foods and animal source foods’. Starch, legumes and vegetables such as cowpea, *moringa* and squash seeds were cited by most groups as part of a healthy diet (Table 2). Some also named *food rich in nutrients* and some mentioned *vitamins*. Their perceptions were that the feeling of eating food you appreciate and that one assumes nutritious form part of a healthy diet. One mother said, ‘What you eat, and you feel good that is eating well’. With regard to growth foods (Table 3), the participants identified protein sources especially fish, legumes, fruits and vegetables as growth foods. They know that common traditional foods can affect the growth of their children. One mother said, ‘Can also prepare vegetable sauce (African eggplant sauce) and put *moringa* in all foods’.

### Socio-cultural perceptions on child care practices

Poor child feeding and care practices are associated with poor child nutritional status [44]. When a child is ill, the first action of the mother is to treat him/her at home with traditional medicine. They go to the hospital only when the child’s condition worsens, after at least 3 days. As this mother from focus group 3 said: ‘We treat at home first, hospital is expensive, and we go there when it is very serious and has worsened’ (see Table 4 for detailed responses). Most mothers said they treat their children at home using tablets or herbal tea and only go to the hospital when the condition worsens. They also said that they get or listen to the advice from the children’s grandmothers on how to care for their children. They said ‘We prepare herbal teas. Grandmothers show us leaves and we prepare. We give as drink or wash them with. The fever goes off quickly’. They primarily give maize porridge to the child during sickness, and when he/she gets better, normal family food is given; their perception of care is linked with feeding the child. Care is comprehensive in that it includes custodial care, such as, supervision, food, shelter and other physical necessities; it goes beyond these to include activities that encourage and aid learning responsible for children’s health, social and psychological needs [45]. Child care supports a child’s emotional, social, intellectual and physical well-being. The discussions in the focus groups only focused on food as part of care when they were probed. This is worrisome if mothers are not aware of their role in child development comprehensively.

### Decision-making and food distribution within households

The way in which food is distributed in the household influences the nutritional status of a child. When distributing meals, especially fish, adults (often the father) are often favoured with the best part [46]. A mother from focus group 4 said: ‘Children are served first and after men are served but the good portion of fish or meat is reserved for the father’. Another mother said: ‘Dad is served first. Dad’s portion is first removed after cooking’. Another one declared: ‘Fathers are outside they could have found something to eat outside, and child would be fasting at home? Children are served first’ (see Table 4 for detailed verbatim responses). Most mothers said children are served first, but the men receive the ‘best’ part of the meals, and in some households, the men are served first, or their portions are removed before serving the children and other members of the household.

### Food taboos and beliefs

Food taboos and misconceptions about food use contribute to the high levels of undernutrition. In fact, it plays a significant role in determining the diets of pregnant and lactating women, infants and young children. In the community studied, there are food taboos, but it is no longer respected by many. Some families or members of certain religions define some food items as appropriate for consumption and others less so [47]. According to the discussion guide used during focus groups, the following were explored: their understanding of food taboos, food taboos they knew restrictions during pregnancy and breastfeeding (women); foods that they ate especially during pregnancy and lactation for good health of the child, foods young children cannot eat according to cultural beliefs and foods not eaten during illness according to cultural beliefs. Their responses are in Table 5. They held food taboos such as not eating pork or dog meat. One of the mothers said: ‘Pork is taboo for Muslims and some families. Others cited dog, cat, catfish, jute sauce with palm oil. There are no explanations, we got it from elders’.

Furthermore, there are dietary restrictions during the period of pregnancy where women avoid nutritious foods for reasons such as avoiding having a big baby, facilitating labour and delivery, or not bearing a child which results in avoidance of certain foods. The study revealed that eggs, cane rat and wild meat were avoided during pregnancy. Long and hard labour was the reason given for avoiding it. They said ‘No eggs during pregnancy. We can eat but whole, we couldn’t share with someone else. A pregnant woman should not prepare eggs and give to someone. She must cook for herself and when she removes the shell it should not have any hurt otherwise, she should not eat because she might have birth complications’. Another group said, ‘When we are pregnant, we do not eat cane rat (giant rats)’. Ekwochi et al. [12] reported similar findings in their study on food taboos and myths in Southeastern Nigeria. Similarly, Gambian women do not eat catfish during pregnancy to prevent giving birth to a dribbling baby [14]. Long and difficult child labour was the main reason given for avoiding those foods [12]. While breastfeeding, mothers eat some foods to have more nutritious milk but avoid other foods such as bananas, papayas and mangoes. These dietary restrictions reduce the potential intake of proteins, vitamins and minerals which are essential for growth and development. These nutritional habits also have an impact on the nutritional status of the child, as the child receives all his/her nutrients from the mother, whether the child is still in the womb or already breastfeeding. In rural Ethiopia, pregnant women avoid eating green leafy vegetables, yoghurt, cheese, sugar cane and green pepper as habitual in fear of obstetric complications associated with the delivery of a bigger infant [13]. David et al. have reported in their study on school-going children in Machakos District, Kenya, that cultural beliefs, taboos and attitudes negatively affect food consumption [48]. In another study in the Gambia, it has been proven that taboos, customs and beliefs contribute to malnutrition among the Fula in different ways [14]. A study in Kenya observed that food taboos are delaying the progress in fighting undernutrition because of cultural beliefs [15]. These beliefs are thought to limit the intake of essential nutrients. Furthermore, a study conducted in Papua New Guinea has shown that many foods that are rich sources of protein have been enlisted as taboos for pregnant women. It is believed that because protein helps the body to grow, if a woman consumes a lot of protein in her pregnancy, then the baby will grow too big leading to complications during labour [16]. It is now believed that some of the food taboos on restrictions on what women could eat are rooted in the patriarchal philosophy of the past rather than physiological requirements. Health professionals responsible for developing nutrition education materials in areas where taboos are still prevalent must be aware of these. They should further ensure coverage and countering those practices that are likely to harm the pregnant mother or the infant.

### Limitations of this study

The FGDs were conducted in *Mina* (local language) since most mothers did not understand French. For analysis, the data were first translated to French and then into English. Quality checks of the *Mina* and English were done by the researcher. However, it is known that the gist of words could have been lost in the progress of transcription and translation.

## Conclusion

The findings revealed that most mothers treated their ill children at home with self-prescribed tablets or herbal tea and only go to the hospital when the condition worsens. Socio-cultural influences were one of the determinants of the decisions that mothers made on child care and feeding practices. Many interventions are focused on the treatment of undernutrition, but it is necessary to raise awareness about the challenges brought by the influence of cultural practices in communities. On the basis of our findings, interventions should be focused on the household dynamics, as cultural norms are a barrier to child care. Educational interventions should be designed to raise awareness of the negative impacts of some socio-cultural practices on the health of the mother and child. It would be beneficial if the authorities organised nutrition education sessions in urban and peri-urban communities to improve the mothers’ knowledge on nutrition, food and child care practices based on local dietary practices. Involvement of men, religious and local authorities, as well as older women (grandmothers), would be an asset in adjusting diets all the while still respecting the culture of the environment.

## Data Availability

The questionnaires and electronic data are available and stored at the Division of Human Nutrition at Stellenbosch University. The data is available upon request from the corresponding author.

## Abbreviations

DHS: Demographic Health Survey
FAO: Food and Agriculture Organization
FDG: Focus group discussion
IQ: Intellectual quotient
SDGs: Sustainable Development Goals
WHO: World Health Organization
